# Experimental Analysis of Space Trusses Using Spacers of Concrete with Steel Fiber and Sisal Fiber

**DOI:** 10.3390/ma13102305

**Published:** 2020-05-16

**Authors:** Welington V. Silva, Ramon Silva, Luciano M. Bezerra, Cleirton A. S. Freitas, Jorge Bonilla

**Affiliations:** 1 Department of Civil and Environmental Engineering, Darcy Ribeiro Campus, University of Brasília, SG12 Building, Brasilia 70910-900, Brazil; welington.vital@gmail.com (W.V.S.); lmbz@unb.br (L.M.B.); 2 Department of Civil Engineering, Federal University of Cariri – UFCA, Av. Tenente Raimundo Rocha S/N, Juazeiro do Norte, Ceará 63048-080, Brazil; andre.freitas@ufca.edu.br; 3 Group for Numerical Methods in Engineering, University of Ciego de Ávila, Ciego de Ávila 65100, Cuba; jorgedbr@unica.cu

**Keywords:** full-scale space truss, steel fiber concrete, sisal fiber concrete, spacer encapsulated with concrete, connection eccentricity, experimental test

## Abstract

Space trusses are structural systems, generally made of tubes, used worldwide because of their advantages in covering long-span roofs. In addition to having a low cost, the truss weight is relatively reduced. The load capacity of these structures depends also on the strength of their node connection. Connections made with the superposition of flattened tube ends trespassed by one bolt are, generally, known as typical nodes. They are inexpensive but present eccentricities that reduce significantly the strength of such space trusses. To increase the truss load capacity, this research presents the results of an experimental program to reduce the eccentricities of the typical nodes. This reduction is done with a new type of spacer made of encapsulated concrete with steel fiber or sisal fiber. The experimental tests showed that the trusses with typical nodes collapsed under reduced load by local failure due to high distortions at the nodes. The trusses with encapsulated concrete spacer showed good results, with an increase in collapse load of 36% and failure by buckling bars.

## 1. Introduction

Space trusses are three-dimensional (3D) reticulate systems used worldwide because of their advantages in covering large, free spaces. In addition to having low cost, the weight itself is relatively reduced. Space trusses are versatile in a range of applications, from small ornamental marquees, the cover of warehouses, gymnasiums, hangars, and shopping centers, to helipads, etc.

Space trusses were copied from nature. The natural elements always seek to minimize stress and maximize strength in an efficient way, taking advantage of the load capacity of all members of the body [1,2]. The natural shapes have exceptional stiffness and use minimum materials to obtain the maximum structural advantage. The natural forms act in the direction of the least force [3].

Humans were quick to copy examples from nature. Examples of three-dimensional modular structures, produced on an industrial scale, were initiated by scientist Alexander Graham Bell [3,4,5,6,7,8,9,10]. In the first decade of the 20th century, he experienced the use of flat trusses in the manufacture of pipes and later with the construction of three-dimensional trusses composed of octahedral and tetrahedral units inspired by the marine shell of Nautilus in an attempt to manufacture light and robust structure to the displacement. In the year 1907, in Canada, Alexander Graham Bell invented what was, probably, the first prefabricated 3D space truss structure.

**Figure 1:**
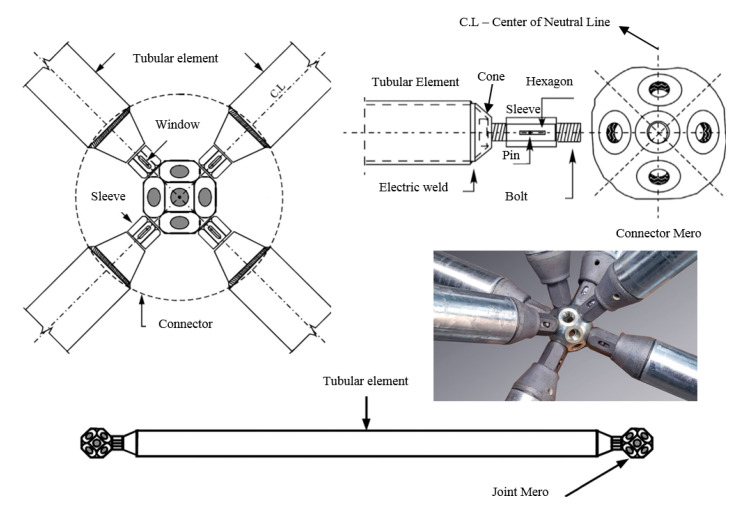
Connection manufactured in 1943, the system more widely used in space trusses in the world.

It was a structure made up of modular tetrahedral elements prefabricated in a factory and attached to the construction site with screws [5]. The structure obtained was raised to form a 30-meter-high observation tower. Despite Bell’s development in the construction of three-dimensional trusses in the early 20th century, they were only used broadly in architecture after the introduction of the *MERO* system in 1943, considered to be a connection of simple execution. It is because Bell’s connection system was complex. *MERO* connection was widely used commercially and was developed in Germany by Dr. Ing. Max Mengeringhausen and Rohbauwwise [1,5,11,12,13,14,15,16,17,18] (see Figure 1).

After the invention of the *MERO* connection, several other systems were patented with the same principle: Steel ball, hexagonal screw, connection sleeve, and hollow section bar. The connection system with spherical nodes showed satisfactory results in experimental tests, mainly because bending moments were not mobilized in the connection. The assembly system is theoretically simple, using only a torque wrench to tighten the bolt [14,19,20,21]. On the other hand, this technology has a high manufacturing cost as a disadvantage. In this way, researchers and designers sought to develop a connection system with a lower cost that could be manufactured in small metallurgical companies [9], and then the typical connection system was developed. 

**Figure 2:**
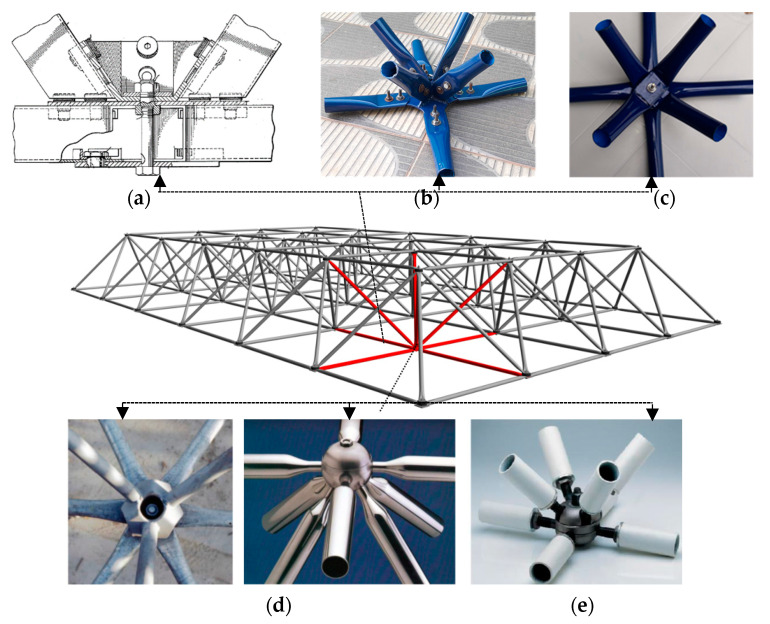
Typical connection models for space trusses. (**a**) Typical connection used in the United States in 1960. (**b**) Connection with flattened steel tube at the ends and bolts in thin-walled plate used in 1968. (**c**) Typical connection with flattened steel tube at the ends and connected with a bolt used in 1968. (**d**) Connections proposed by Du Château in 1970. (**e**) *VESTRUT* system patented connection space in 1980.

In this context, the first typical node connection used as an economic alternative in the project was in 1960 in the United States by Richard Fuller and Konrad Wachsmann in the construction of a hangar cover at London airport (see Figure 2a). Later in Brazil in 1968, the roof of the Anhembi Exhibition Park was built, in the city of São Paulo (see Figure 2b) with typical connections. It became the largest roof in the world in aluminum using space truss. The space structure covered 70,000 square meters. Later in 1970 in France, engineer Stéphane Du Château developed also two alternative connections systems for space trusses [22] (see Figure 2d). In Italy, in 1980, the *VESTRUT* system was widely used as a space truss connection option [23] (see Figure 2e).

The assembly of the space trusses with typical connections uses the overlapping of stamped bar ends joined by a single trespassing bolt. Such connections, generally, go against the safety recommendations of design codes of using more than one bolt. This system presents several structural problems; one of them is due to the eccentricities in the typical node. Eccentricity generates bending moment, increase in stresses, and produces the failure of the connection at reduced load, therefore, with an inefficient use of the resistant capacity of the bars (see Figure 2c).

As shown, the first records of the use of space trusses applied as a roofing system date from the beginning of the 20th century [7,24]. However, in the following years, several accidents were recorded with space trusses, due to the progressive collapse of the connections [8,25,26,27,28,29,30]. 

## 2. Collapses in Space Truss and Eccentricity Correction

**Figure 3:**
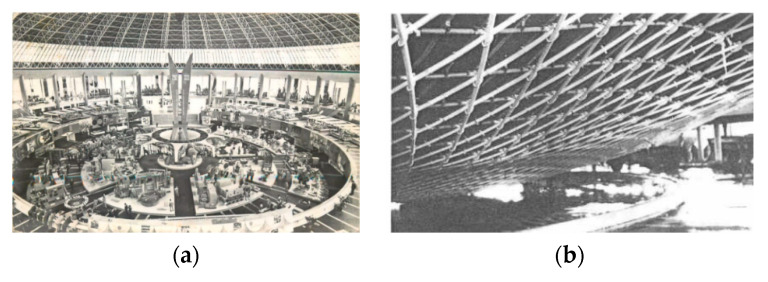
Mechanism of collapse of the Bucharest, Romania, cover in 1963 [32,39], (**a**) inside view before collapse, (**b**) inside view after collapse.

The first large lattice structure to collapse was the Bucharest Dome built in 1961 [31,32,33,34]. The building known as the national economy exhibition pavilion in Bucharest, Romania, was designed by Ferdinand Lederer [35]. It collapsed in 1963, less than two years [33,36] after the inauguration. The roof was a symmetrical dome composed of 26 bands of 3D trusses. The structure was used to contour a 65-m radius hubcap surface. [32,35,37,38]. Figure 3 shows details of the roof before and after the collapse that occurred in the welded connections.

**Figure 4:**
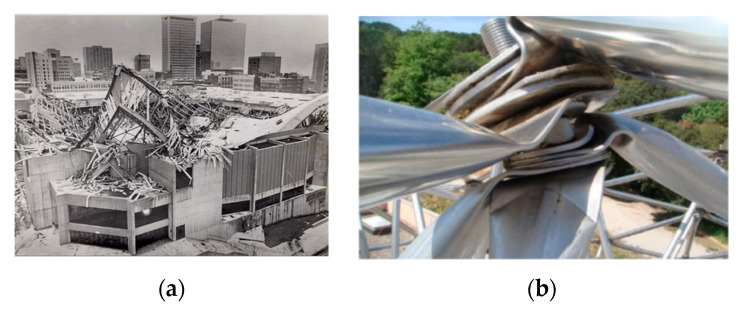
Collapse detail with space truss, (**a**) details of the collapse of the Hartford Civic Center Coliseum [25], (**b**) cover collapse after a snowfall in Gerona, Spain [49].

A coliseum in the United States collapsed [25,26,40,41,42,43,44,45,46] after various shows and games. Its construction began in 1960 [40] and was one of the major projects in the city of Hartford at the time, being completed in 1973. The 3D lattice roof was shaped like pyramids, with dimensions of 9.40 m by 9.40 m and a height of 6.45 m. The roof was modulated with 12 pyramids in one direction and 10 in the other one, comprising the size of 112.80 m by 94.0 m. In the 1980s, several investigations were carried out to identify the causes of the accident [25,26,29,35,43,47,48]. Figure 4a shows the roof after the collapse.

In the province of Gerona in Spain, the cover of a sports center, a multisport gymnasium, collapsed. The roof dimensions were 50 m × 30 m [49]. The connection of the double layer mesh was the typical node system with stamped ends; the work was completed in 2000. However, in 2010, due to snow overload, the structure collapsed, as a consequence of plenty of rotations in the typical connections due to the eccentricities contained in the connection, as shown in Figure 4b.

**Figure 5:**
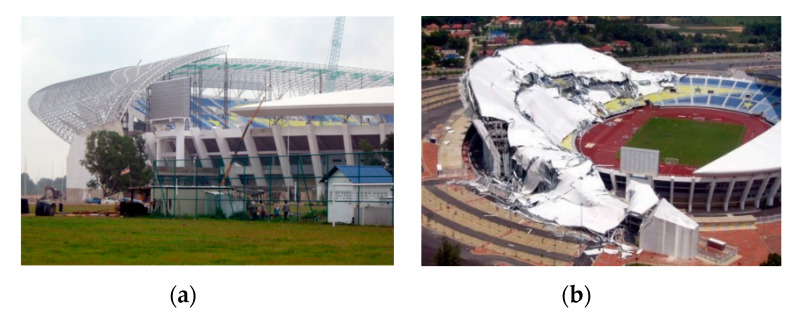
Detail of the collapse of the football stadium roof in Malaysia [52], (**a**) details of the construction of the Shah Alam Stadium at Malaysia [5], (**b**) detail of the roof collapse of the Shah Alam Stadium at Malaysia [35,50].

In Malaysia, another type of collapse in space truss structure occurred in the city of Terengganu, which had a double-layer structure, with a capacity for 50,000 soccer fans. The stadium cover collapsed on June 2, 2009 (see Figure 5) [50,51].

**Figure 6:**
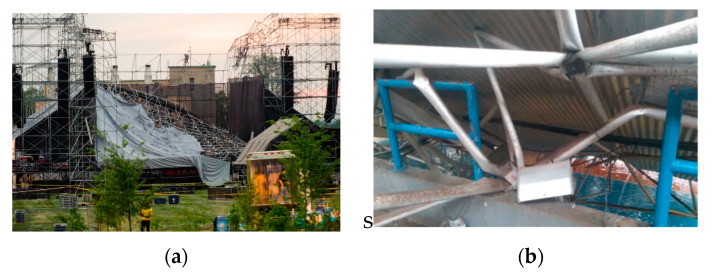
Connection failure and space truss rupture, (**a**) detail of the failure in typical connection at city of Ontario, Canada, (**b**) gymnasium roof collapse, Brazil [60].

In 2012, in the city of Ontario, Canada, in Downsview Park, there was a collapse of a 3D lattice structure; the park stage collapsed [53]. The connection used was a typical node connection. Figure 6a shows the details of this collapse due to welding failures. In Brazil, accidents also happened in 3D structures [9,17,24,54,55,56,57,58,59]. One major accident was the roof of the Manaus Convention Center in April 1994. The most recent was the collapse of the Georgiano Gymnasium in March 2019, after a strong rain. This facility had a capacity for 6500 fans, with dimensions of 120 m by 65 m; the lattice structure collapsed. Figure 6b shows the node collapse of this gymnasium.

Several other collapses in 3D trusses have been observed in the world. However, the ranges of space truss structures have been reevaluated and some have been strengthened in recent years [61,62,63,64,65,66,67,68,69,70], thanks to increasingly robust computer systems and commercial Finite Element Method (FEM) programs and complex nonlinear analyses [10,30,56,71,72,73,74,75,76,77,78,79,80,81,82].

In summary, the motivation of this paper was related to the fact that inexpensive but common 3D trusses present local failure at their connections, specifically in stamped typical connections, which deform excessively causing truss collapse [83]. This problem is explained by the geometric changes generated in typical connections [84] due to the flattened ends of the truss bars and tubes [4,85,86]. The increasing stresses, at the stamped ends, reduce the truss load capacity [49,55,87].

### 2.1. Correction of Eccentricity in Typical Connections

**Figure 7:**
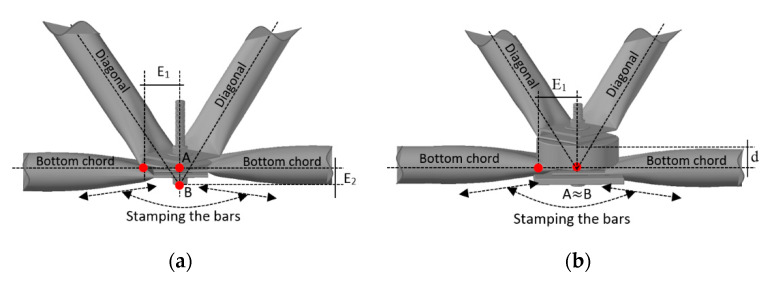
Proposal to correct the eccentricity [9], (**a**) typical connection with eccentricity, (**b**) correction of eccentricity “*d = E_2_*”in the connection.

Many space truss accidents involving typical connections are characterized by a progressive collapse triggered by the local collapse of the connection, not to the ultimate buckling of the truss bars. The collapses involving typical connections were a consequence of the stamped tubes overlapped and interconnected with a single bolt. This produces the incompatibility of lines passing through the center of gravity of the bars arranged in the diagonals and on the truss edges (see Figure 7). Consequently, it generates eccentricities (see Figure 7a). This situation differs from the nodes idealized in a theoretical truss, and, above all, from the assumptions adopted in most design offices. It is possible to observe that there are two eccentricities: *E_1_*, horizontal (stamped region) and *E_2_*, vertical (distance between points A and B (see Figure 7)). The proposal, developed by [59], was to correct the eccentricity (*E_2_*), using a spacer element. In this case, it is observed that the eccentricity correction *E_2_* is made using an element that provides the distance *d* equal to *E_2_* between the diagonals and the bottom chord. Thus, points A and B (Figure 7a) can be considered superimposed (Figure 7b).

**Figure 8:**
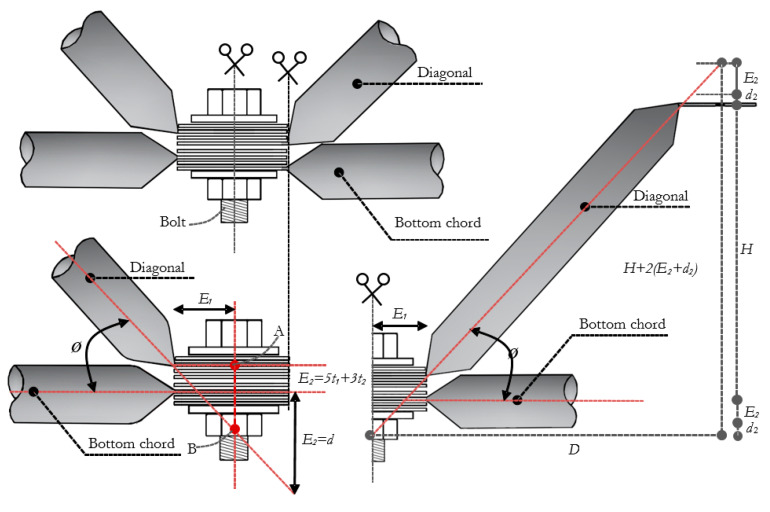
Trigonometric relations for typical connection in space truss.

The vertical eccentricity *E_2_* is proportional to the angle *ϕ*, being equivalent to the correction distance *d*. Figure 8 shows that the measurement of *d_2_* is equal to *5t_1_* of the pipe wall thickness (flange) *t_1_* plus *3t_2_* of the pipe wall thickness of the diagonal *t_2_*. Considering the wall thicknesses of the same tubes (diagonals and chords), we have a total of *8t*.

Now, trigonometric relations to the two triangles formed in Figure 8 can be applied. The first triangle is obtained using the *E_1_* side, and the second triangle composed by the base of the *D* side. Equating the two triangles, Equation (1) is obtained.

(1) $$d=\frac{2HE1}{l\sqrt{2}-4E1}-8t$$

where: *E_1_* Eccentricity due to flattening of the bar; *t_1_* and t_2_ is the thickness of the tube; *H* represents the height of the truss; *L* the length of the chord; *d* is the spacer thickness necessary to correct the eccentricity *E_2_*. However, the use of this equation requires that the spacer has sufficient resistance to support the compression force. Thus, concrete is a candidate to be used as a spacer due to its good resistance to compression. Nevertheless, to avoid concrete cracks, two fiber mix options were evaluated. One option was concrete mixed with steel fiber and the other was concrete mixed with sisal fiber. Both concrete mixtures can be encapsulated by cold-formed steel profiles to restrain concrete deformation. The concrete spacers have the function to eliminate eccentricity *E_2_* (Figure 7) and prevent the appearance of bending moments in the connections.

### 2.2. Application of Fiber-Reinforced Concrete 

Conventional concrete still has some deficiencies such as low ductility, plastic shrinkage, and small tension strength. Thus, fiber additions to concrete is common practice, which seeks to reduce the cracks, improving performance to tensions’ forces. The fibers act as connection bridges, transferring the stresses to another side of the concrete matrix, and minimizing stresses at the ends of the cracks. The use of discrete fibers is one effective solution to reinforce the matrix for improving the tensile and flexural performances of the plain concrete. Among the fibers, steel fiber was one of the earliest and it is one of the most effective materials for improving the mechanical properties and impact resistance of concrete [88]. The first structural use of steel fiber-reinforced concrete was in 1971 for the production of collapsible panels for a London Heathrow Airport parking garage. Since then, this type of concrete reinforcement has gained much interest in the construction industry, and also among researchers. Table 1 presents briefly some applications of reinforcement with the addition of fiber to the concrete.

The use of fiber-reinforced concrete has a better deformation capacity, impact resistance, energy absorption, and tensile strength. This paper proposes to correct the eccentricity of 3D trusses using concrete with fiber as spacers using two types of fibers: Steel fiber and sisal fiber.

## 3. Experimental Program and Methods

### 3.1. Materials Used for Construction of the Spacers

For the construction of the spacers, a composite cement Portland CPIII-40, from the *APODI* Company was used. This cement was tested and checked. The granulometry test showed that the CPIII-40 granulometric distribution obtained was 95% and the cement grains were smaller than 50 µm, with 55% being less than 20 µm. The fine aggregate used in the production of the concretes was a quartz river sand. This sand has the following characterization: A maximum diameter of 4.80 mm, a fineness modulus of 2.7, natural moisture of 0.72%, density of 2.6 g/cm³, and water absorption of 1.19%. The coarse aggregate used was basaltic gravel, with a lamellar shape, the values of a specific mass, water absorption, and the Los Angeles abrasion test, and granulometry obtained the following results: Maximum aggregate diameter of 9.50 mm, fineness modulus of 5.93, natural humidity of 1.57%, density of 2.7 g/cm³, and water absorption of 25%.

### 3.2. Steel Fiber and Sisal Fiber

The new steel fibers presented in this paper were produced from steel wires with tensile strength greater the 1000 MPa, manufactured by Dramix® from the company ArcelorMittal Corporation in the city of Ipatinga, Brazil. For the manufacture of the new steel fibers, a simple tool was used to cut and bend. The new fiber had a straight section with 25 mm of the length, plus two inclined anchors, in the shape of a hook at the ends of the fibers. The hook size was 10 mm, so that the total length of the fiber was 45 mm, similar to the material used in the fiber of the model Dramix® ZC45/0.5. 

**Figure 9:**
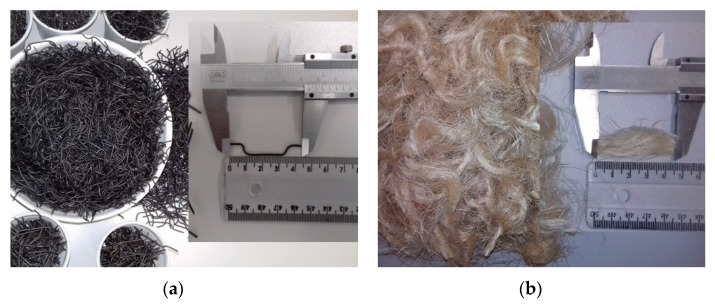
(**a**) Hooked-end steel fibers, (**b**) sisal fibers.

In the context of the civil construction environmental impact, fibers from natural resources, such as sisal fiber, appear as an alternative to synthetic fibers. However, the elements incorporating such technology are still on the market [102]. The sisal fibers used in this work were extracted from Agave Sisalana plant by a process called decortication. The fibers were received in bundles of long fibers, approximately 1 meter long. Before cutting them into segments of 45 mm, it was necessary to process them to remove impurities. The fibers were submerged into water at 70 ± 5 °C for approximately one hour. After this process, the fibers were air-dried for 48 h and then manually cut. The steel fiber and sisal fiber, inserted in the concrete matrix, were used with 1% of the concrete volume fraction (V_f_ = 1%) [103,104,105,106]. The strength of the steel wire was tested before cutting and bending. All tests for material and mechanical properties’ characterization were done in the Structural Laboratory and in the Materials Laboratory, both at the Federal University of Cariri (UFCA). Table 2 and Table 3 give the properties of the steel and sisal fibers. Figure 9 shows pictures of these fibers.

Table 4 presents the compositions and abbreviated names of the three concrete mixes, with water cement ratio of 0.5, used for the manufacturing of the spacers.

**Figure 10:**
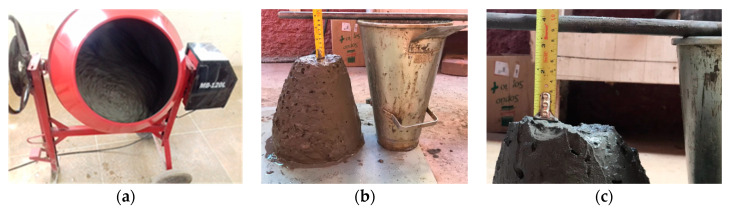
Execution of concrete mixes. (**a**) Inclined-axis electric concrete mixer, (**b**) concrete without fiber, (**c**) concrete with using fiber.

To obtain a homogenous concrete mix with sufficient workability, all batching was done by weight. The concrete was mixed with an electrical mixer (see Figure 10a). The interior surface of the mixer was cleaned and moistened before placing the materials. Both coarse and fine aggregate was placed and mixed for several minutes into the mixer after the cement was added. 

The materials were mixed until a uniform color was obtained. After that, half of the water quantity was added and mixed for several minutes, too. After 5 min beating, finally the rest of the water quantity was added to the mixture and mixed for about 3 min. When steel fiber and sisal fiber were added to the mix, they were uniformly distributed in the layer top of the mixer.

After mixing, the workability test was carried out with a slump test before the fresh concrete was poured into the spacer molds. The average workability of concrete without fiber and with fiber was around 10 cm (see Figure 10b,c). The concrete mixture was poured into the workability test molds in two layers. When each layer was completed, the sides of the molds were hammered by a rubber driver, to shake the mix and consolidate the layer into the molds. Then the concrete mixture was compacted using a compact table vibrator. The compactness took approximately 40 s for each layer. During compaction, air bubbles appeared on the surface as an indication that the unwanted air was taken away. 

After that, the surface of the concrete was leveled off. Then, the specimens were covered to prevent water evaporation. After 24 h, the specimens were taken from the molds and placed for 7 days in a tank, and then for 28 days cured in water with 23 °C temperature and humidity of 76%.

### 3.3. Experimental Test of the Concrete

This study performed a four-point bending test for the determination of the tension softening properties and curves of concrete with and without fiber. For each concrete mix, shown in Table 3, three beam specimens were tested. The beam fabrication and dimensions (width, height, and length, respectively, 100 mm × 100 mm × 400 mm) followed the recommendations of UNI-11039 [107] or RILEM TC162-TDF (2003) [108].

**Figure 11:**
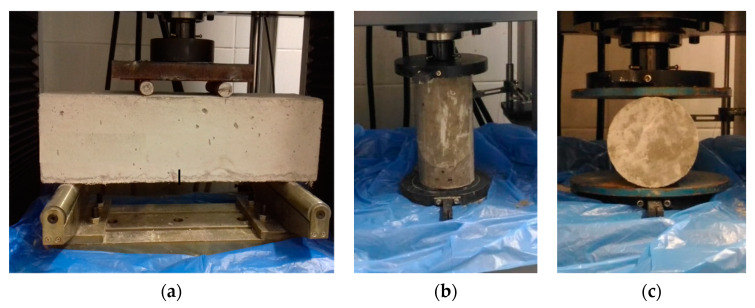
(**a**) Four-point bending test, (**b**) compression test, (**c**) tension indirect test.

After the completion cure of concrete and prior to the test, each beam specimen was cut with a notch located at mid-length of the beam. The notch was set with a constant width of 4 mm (see Figure 11a). A WDW-300KN-UTMs computer control electronic universal testing machine with a capacity of 2000 kN was used for the four-point bending test. Load was applied under displacement control at a speed of 1/1500 of the specimen span length (133 mm) per minute. One Linear Variable Differential Transformer (LVDT) with a capacity of 100 mm was used to measure the deflection of the center of the beam specimen during the test. A clip gage was attached at the bottom of the specimen to measure the crack width at the notch. To determine the compressive strength of concretes, 27 specimens were molded with 9 specimens for each type of concrete, following the guidelines of [109], with dimensions of 100 mm of diameter and 200 mm of height (see Figure 11b). To obtain the tensile strength of concrete, 9 specimens were molded, with 3 specimens for each type of concrete. The tensile strength was obtained by means of the tensile test by diametrical compression, according to [110]. Figure 11 illustrates the specimens for the characteristics of tension and compression concrete properties.

**Figure 12:**
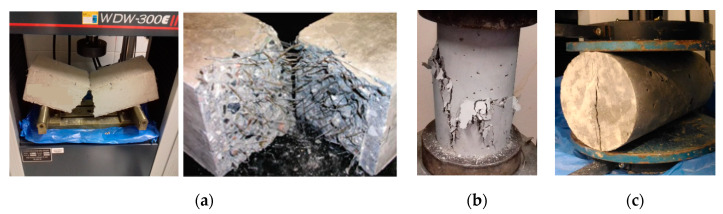
Result of experimental tests. (**a**) Four-point bending beam test, (**b**) compression test, (**c**) tensile indirect test.

Figure 12 shows a typical failure configuration after the four-point bending test, compression strength, and tension strength. After failure, it was shown that one large crack existed, accompanying fibers, which played an important role in bridging two crack faces.

**Figure 13:**
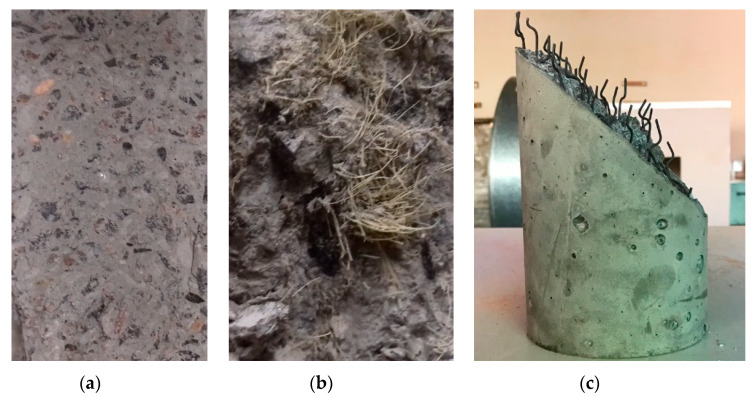
Concrete types. (**a**) Concrete without fiber, (**b**) concrete with sisal fiber, (**c**) concrete with new steel fiber.

**Figure 14:**
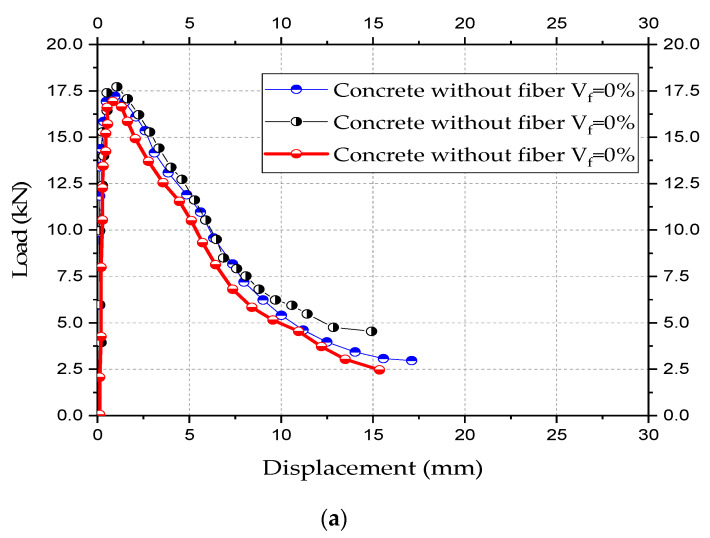

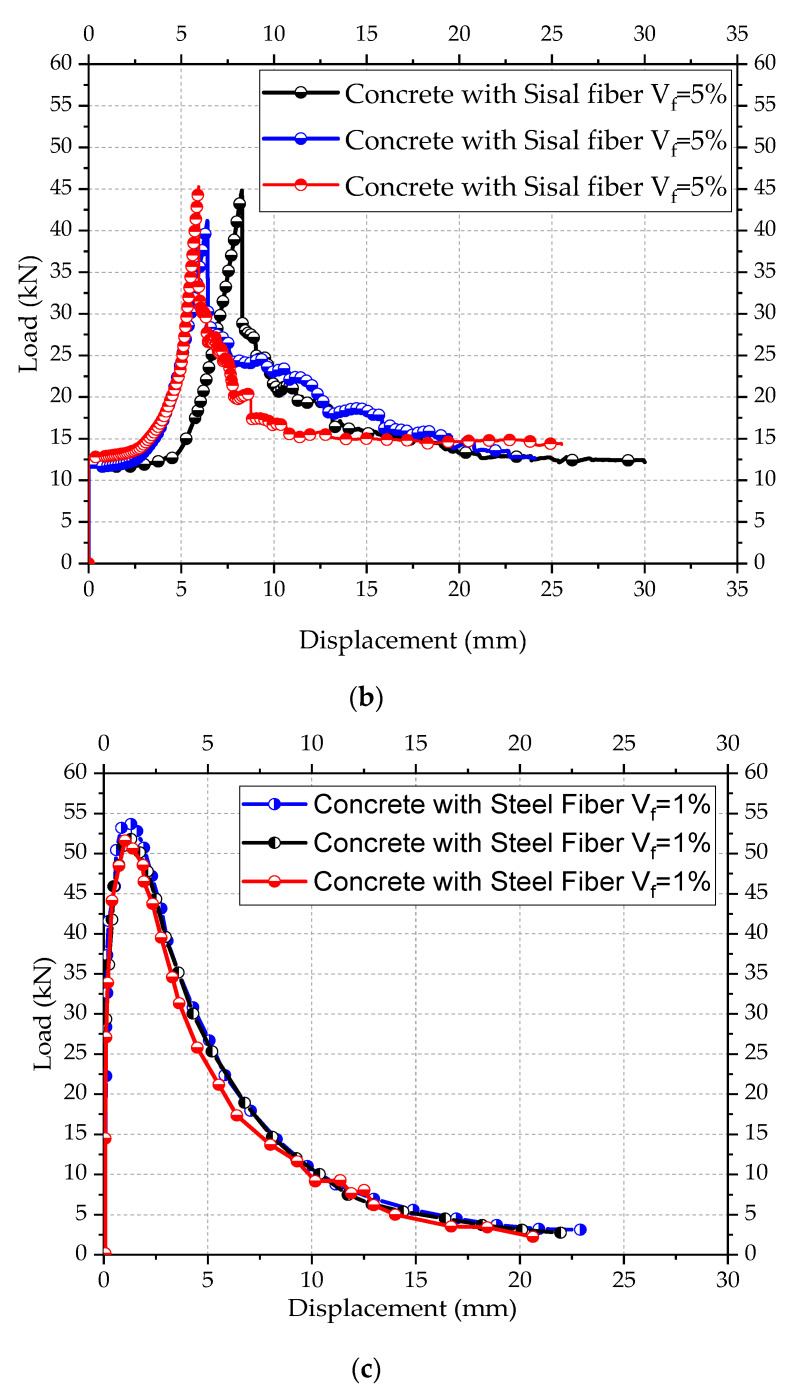
Results of experimental tests. (**a**) Four-point bending beam test, (**b**) compression test, (**c**) tensile indirect test.

Due to the bridging mechanism of fibers, concrete with fiber can provide superior performance, especially under tension, as compared to concrete without fibers. Figure 13 presents the types of concrete tested. Figure 14 shows the experimentally obtained load-displacement curves.

The mechanical properties of the reinforced concrete with steel and sisal fibers are in Table 5.

**Figure 15:**
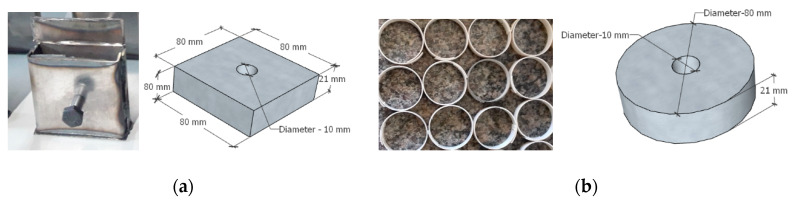
Shape of the spacers used in the space truss. (**a**) Shape of the cold-formed steel with 1.5 mm thickness for encapsulating of the concrete with steel fiber, (**b**) Polyvinyl Chloride (PVC) molds for spacers with concrete with steel fiber without the encapsulation of concrete.

It was observed that for the three types of concrete tested the one with steel fiber presented the best behavior in the bending and compression tests. The analysis of the tenacity showed that concrete with steel fibers reinforcement presented a superior behavior compared to the concrete with sisal fiber and without fiber. However, the concrete with sisal fiber showed workability difficulties during the execution of the specimens. In this way, the spacers were built with concrete with steel fiber. For this, two spacers were built: One just with the concrete with steel fiber and the other one concrete with steel fiber but encapsulated by a steel cold-formed profile. Figure 15 presents details of the two spacers.

### 3.4. Procedures and Instrumentation of the Space Truss Experiments

**Figure 16:**
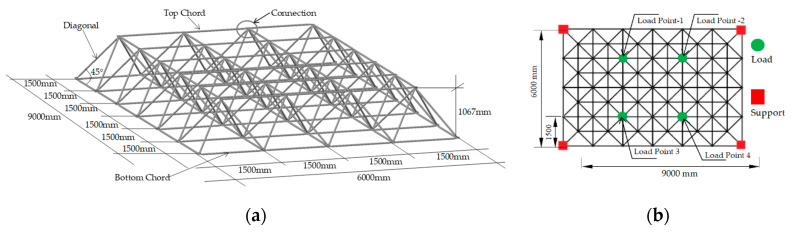
Space trusses tested: (**a**) Dimensions and (**b**) plan view of the prototypes.

**Figure 17:**
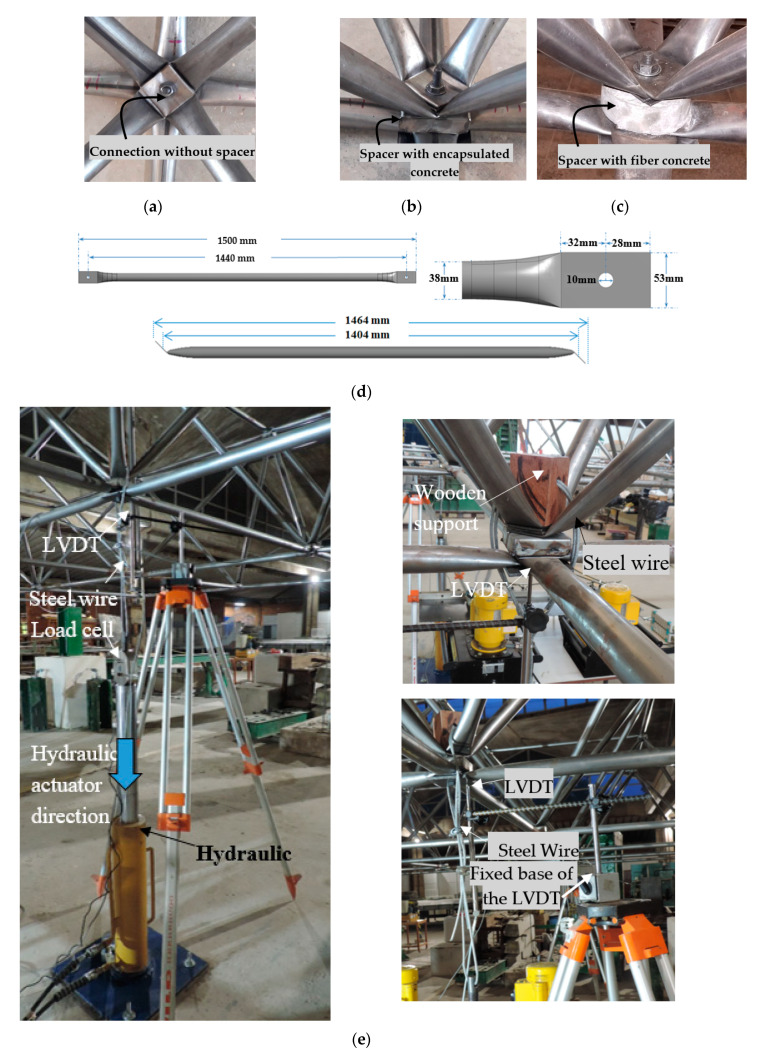

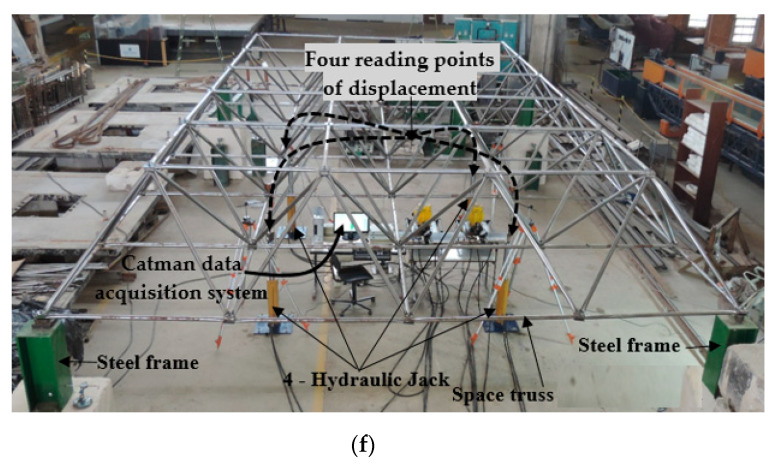
Detail of prototypes: (**a**) Space truss with typical connection without spacer, (**b**) space truss with spacers made of encapsulated concrete with steel fiber, (**c**) space truss with spacers made of concrete with steel fiber, but not encapsulated, (**d**) length of the steel bars of the space trusses, (**e**,**f**) details of the instrumentation used.

To test the strength of space trusses using spacers compared to space trusses without spacers, five specimens with dimensions of 9000 mm by 6000 mm and height of 1067 mm were built and tested in the Structures Laboratory of the University of Brasilia (UnB). Each truss specimen, with spacers applied at each typical node for correcting eccentricity *E_2_* (see Figure 8), used 56 spacers. Two specimens did not use spacers, and used just the typical node connections. One specimen used spacers made just of concrete with steel fibers but without the encapsulation of the concrete. Two specimens used encapsulated concrete spacers with concrete with steel fibers. Figure 16 shows the details of the prototypes tested. Figure 17 shows the dimensions of the elements of the space truss, with details of the instrumentation and of the experimental devices used. All structures were assembled following a sequence of bars to keep the space truss pattern and symmetry. Figure 17 shows some details of the lab experiments. All prototypes were initially prepared on the laboratory floor and then lifted to a short steel column (Figure 17f). To standardize the tightening of the bolt, all bolts were tightened with a 50 Nm. The data were obtained by reading the loads in the HMB Catman panel using the load cells initially calibrated and the LVDT disposed in four points (Figure 17e), where the forces were applied, see Figure 16b and Figure 17f. The direction of the application of the loads (Figure 16b) occurred in the same direction of the gravitational force, so that the structure was pulled down by the hydraulic jacks that were fixed on the reaction floor of the laboratory.

## 4. Results of Experimental Tests

The five experimental tests evaluated the load capacity of the space truss, using 4 LVDT with a progressive load rate of 0.20 kN/min and using the HBM Catman system. The five tests compared displacements and collapse loads of space trusses. Two experiments were done with trusses with typical connection without spacer. Two experiments were done with space trusses with typical connection corrected with spacers made of encapsulated concrete with steel fiber, and two experiments were done with spacers with the same concrete with steel fiber but not encapsulated.

**Figure 18:**
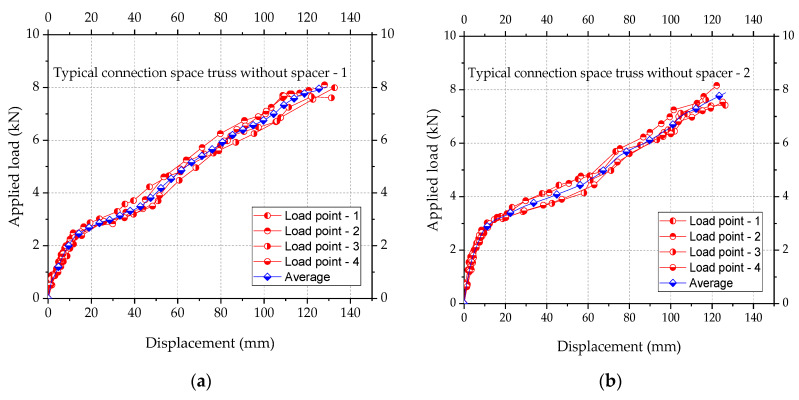

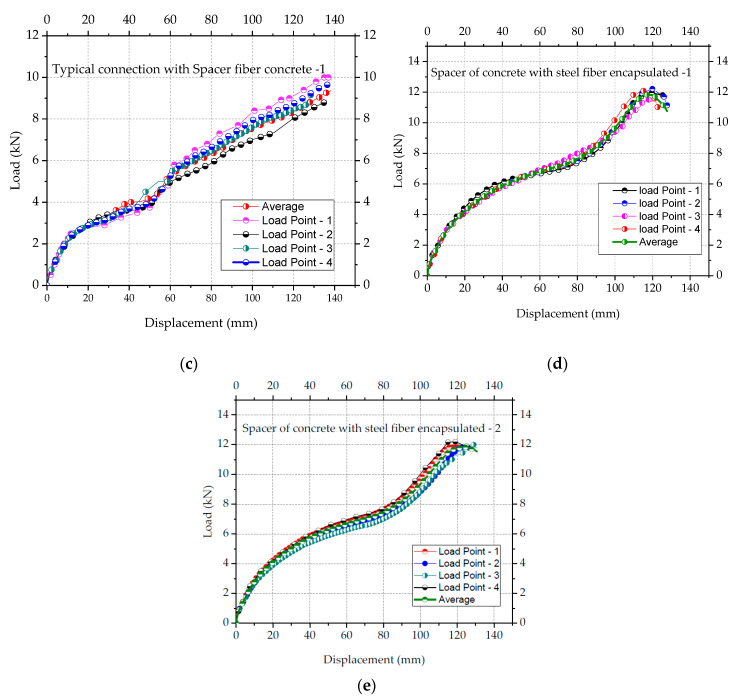
Result of the experimental tests. (**a**,**b**) Space truss without spacer, (**c**) typical connection with spacer of concrete with steel fiber, (**d**,**e**) space truss with spacer of concrete with steel fiber encapsulated with cold-formed steel.

**Figure 19:**
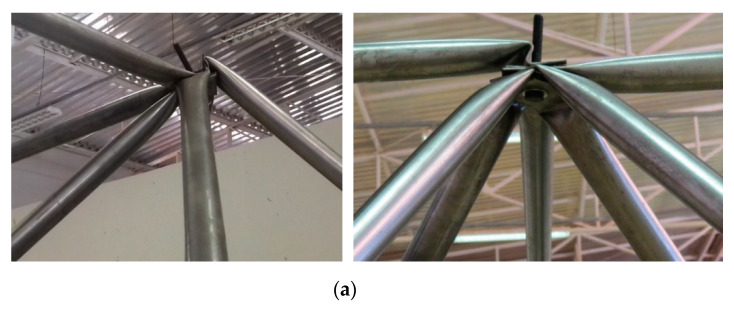

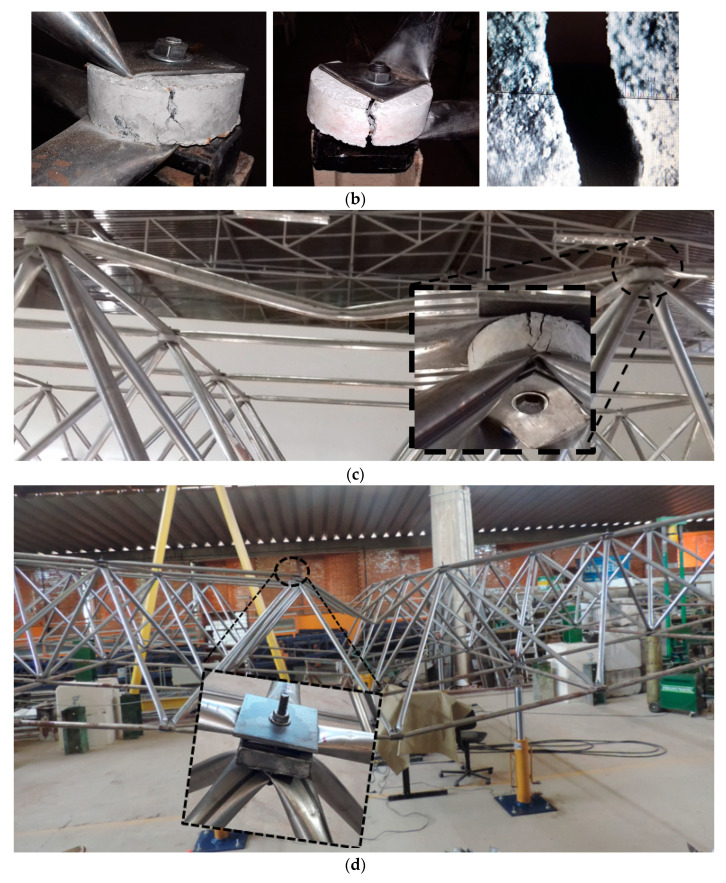
Details of the space truss collapse modes. (**a**) Collapse of typical connection caused by bending moment due to eccentricities, (**b**,**c**) failure of the concrete spacer, (**d**) concrete encapsulated with steel fiber.

Porotypes with typical nodes without any spacer to correct the eccentricity *E2* (Figure 8) collapsed with a reduced load compared with the other prototypes with spacers. It was noticed that the use of spacers avoided local distortions of the nodes of the trusses. Typical connection with spacer made of encapsulated concrete with steel fiber achieved the total design capacity of the tubes. In these cases, the tests led some tubes to the expected global buckling limit. The use of spacer with steel fiber-reinforced concrete but not encapsulated presented a collapse load higher than the trusses without spacers, but smaller than the load capacity of the trusses with the encapsulated concrete. Figure 18 and Figure 19 show details of all the tests.

The experimental results of the five tests were plotted in Figure 18 and Figure 19 with the details of the shapes of the trusses after the collapse. Figure 18 shows the results for each prototype tested. Such results are in terms of applied loads vs. displacements at the nodes where loads were applied (See Figure 16b). When using spacers, collapses took place by buckling of the bars and tubes, while in the trusses without spacers collapse took place by high local distortions of the nodes. In prototype trusses with spacers, no significant distortions at the nodes were observed. The experimental results of the five tests were plotted in Figure 18 and Figure 19 with the details of the shapes of the trusses after the collapse. Figure 18 shows the results for each prototype tested. 

Such results are in terms of applied loads vs. displacements at the nodes where loads were applied (See Figure 16b).

When using spacers, collapses took place by buckling of the bars and tubes, while in the trusses without spacers collapse took place by high local distortions of the nodes. Only one truss was tested with spacers made of concrete with steel fiber without the encapsulation of the concrete. In this case, it was observed that the spacer did not perform well compared with spacers with concrete encapsulated. The results showed that typical connection without spacers presented the largest local distortions and deformation, too. In this case, the collapse load average was 7.75 kN, corresponding to 128 mm of average vertical displacement. For the prototypes with spacers with encapsulated concrete, the average displacement was 120 mm with the average collapse load of 12.2 kN. Moreover, for the prototypes with spacers with fiber concrete without encapsulation of concrete, collapses took place at an average load of 9.70 kN and an average displacement of 132 mm due to failure of the spacer of concrete.

## 5. Conclusions

Connections made with the superposition of flattened tube ends trespassed by one bolt are known as typical connections or typical nodes. Trusses with typical connections are widely used in many countries. They are inexpensive and easy to assemble. However, they offer a risk of collapse due to the eccentricities in the connection. Many types of collapse in space trusses over the years have been reported in the literature. A significant number of them took place at the connections. Space trusses with typical connection present eccentricities. These eccentricities, due to the end-flattened tubes, produce bending at the typical nodes and reduction of bending stiffness of the tube ends. Typical connection collapses do not achieve the compressive bearing load capacity, i.e., the buckling limit load of the tubes. 

Typical connections collapse is characterized by excessive distortion at the nodes. This work reported the results of an experimental program devoted to space trusses with corrections for the typical connections. In this research, the use of spacers increased significantly the load capacity of the typical connections. The height and dimension of the spacers were treated in this research, too. A new type of spacer made of encapsulated concrete was presented. Concrete was encapsulated with cold-formed steel profiles. Two types of encapsulated concrete were developed: Concrete mixed with steel fiber and concrete mixed with sisal fiber. The first option showed much better mechanical properties than the alternative sisal fiber. In that sense, five space truss prototypes tests were performed: (1) Two trusses with typical connection and no correction of the eccentricities, (2) one truss using spacers of concrete not encapsulated, just with steel fiber concrete, (3) two trusses with typical connection using spacer of encapsulated concrete mixed with steel fiber. The tests showed that:

- Spacers can efficiently prevent the premature collapse of space trusses with typical connections,
- The use of spacers of the concrete mixed with steel fiber not encapsulated did not resist the high stress in the connection and radial cracks were observed at failure, and
- Space trusses with spacers of encapsulated concrete mixed with steel fiber showed 36% greater load capacity compared to space trusses with typical connections with no eccentricity correction.

## Figures and Tables

**Table 1:** Some fiber concrete applications.

Main Uses of Fiber in Concrete	References
Avoid cracking on industrial floors and pavements;	[89]
Application in tunnel coverings with shotcrete;	[90]
Prefabricated elements such as concrete tubes and thin-walled concrete;	[91]
Structural reinforcement of concrete plates with staple fibers and randomly distributed in the concrete matrix;	[92,93]
Behavior of fiber reinforced concrete for controlling the rate of cracking in canal-lining;	[94]
Application of fibers in reinforced concrete of columns to improve resistance to seismic actions;	[95]
Reinforcement of mortars for masonry execution;	[96]
Reinforcement of reinforced concrete beams;	[97,98]
Addition of fiber to improve the toughness of the concrete to exposure;	[99,100]
Use of mortar reinforced with steel fiber for seismic reinforcement.	[101]

**Table 2:** Properties of steel fiber.

Commercial Name	Configuration	Property	Specifications	
			Values	*cov*
New steel fiber	Hooked Ends 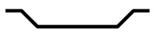	Density	7860 kg/m³	-
		Ultimate Strength	1130 MPa	0.048
		Modulus of Elasticity	$200\times 10^{3}$ MPa	0.077
		Strain at proportion limit	$5650\times 10^{-6}$	-
		Poisson´s ratio	0.28	-
		Average Length-*L_f_*	45 mm	-
		Nominal Diameter-*D_f_*	0.50 mm	-
		Aspect ratio (*L_f_/D_f_*)	90	-

**Table 3:** Properties of sisal fiber.

Property of the Sisal Fiber	Values	
	Minimum - Maximum	*cov*
Length (mm)	45 mm	-
Density (g/cm³)	0.75–1.07	0.021
Tensile strength (MPa)	227.80–1002.30	0.182
Modulus of Elasticity (GPa)	10.94–26.70	0.193
Failure deformation (%)	2.08–4.18	0.175
Water absorption to saturation (%)	180.00–250.00	0.064
Diameter (mm)	0.08–0.030	0.185

**Table 4:** Dosage used in the experimental procedure for 1 m³ of concrete.

Nomenclature	Trace by Weight	Cement *APODI* (kg/m³)	Sand (kg/m³)	Coarse Aggregate (kg/m³)	Water (*l*)	Fiber (kg/m³) or (V_f_ = 1%)
C30	1:2.5:2.34:0.50	351.26	878.15	821.94	175.63	-
C30SteelFiber	1:2.5:2.34:0.50	351.26	878.15	821.94	175.63	78.0 (1%)
C30SisalFiber	1:2.5:2.34:0.50	351.26	878.15	821.94	175.63	14.52 (1%)

**Table 5:** Concrete properties.

Concrete Specimen’s	Compressive Strength		Splitting Tension Strength		Flexural Tension Strength		Modulus of Elasticity	
	(MPa)	*cov*	(MPa)	*cov*	(MPa)	*cov*	(GPa)	*cov*
C30	29.87	0.46	1.62	0.018	3.67	0.020	19.79	1.53
C30SteelFiber	31.89	0.66	2.74	0.064	3.84	0.074	20.17	1.84
C30SisalFiber	27.06	1.69	2.18	0.187	3.30	0.172	17.32	2.28
